# Decoding the RNA viromes in rodent lungs provides new insight into the origin and evolutionary patterns of rodent-borne pathogens in Mainland Southeast Asia

**DOI:** 10.1186/s40168-020-00965-z

**Published:** 2021-01-21

**Authors:** Zhiqiang Wu, Yelin Han, Bo Liu, Hongying Li, Guangjian Zhu, Alice Latinne, Jie Dong, Lilin Sun, Haoxiang Su, Liguo Liu, Jiang Du, Siyu Zhou, Mingxing Chen, Anamika Kritiyakan, Sathaporn Jittapalapong, Kittipong Chaisiri, Phillipe Buchy, Veasna Duong, Jian Yang, Jinyong Jiang, Xiang Xu, Hongning Zhou, Fan Yang, David M. Irwin, Serge Morand, Peter Daszak, Jianwei Wang, Qi Jin

**Affiliations:** 1 NHC Key Laboratory of Systems Biology of Pathogens, Institute of Pathogen Biology, Chinese Academy of Medical Sciences & Peking Union Medical College, Beijing, PR China; 2 Key Laboratory of Respiratory Disease Pathogenomics, Chinese Academy of Medical Sciences & Peking Union Medical College, Beijing, PR China; 3 EcoHealth Alliance, New York, NY USA; 4 Wildlife Conservation Society, Viet Nam Country Program, Ha Noi, Vietnam; 5 Wildlife Conservation Society, Health Program, Bronx, NY USA; 6 Faculty of Veterinary Technology, Kasetsart University, Bangkok, Thailand; 7 Faculty of Tropical Medicine, Mahidol University, Bangkok, Thailand; 8 GlaxoSmithKline Vaccines, Singapore City, Singapore; 9 Virology Unit, Institut Pasteur in Cambodia, Phnom Penh, Cambodia; 10 Yunnan Institute of Parasitic Diseases, Pu’er, PR China; 11 Department of Laboratory Medicine and Pathobiology, University of Toronto, Toronto, Canada

**Keywords:** Mainland Southeast Asia, Rodent lungs, Core virome, Viral evolution, Emerging infectious diseases

## Abstract

**Background:**

As the largest group of mammalian species, which are also widely distributed all over the world, rodents are the natural reservoirs for many diverse zoonotic viruses. A comprehensive understanding of the core virome of diverse rodents should therefore assist in efforts to reduce the risk of future emergence or re-emergence of rodent-borne zoonotic pathogens.

**Results:**

This study aimed to describe the viral range that could be detected in the lungs of rodents from Mainland Southeast Asia. Lung samples were collected from 3284 rodents and insectivores of the orders Rodentia, Scandentia, and Eulipotyphla in eighteen provinces of Thailand, Lao PDR, and Cambodia throughout 2006–2018. Meta-transcriptomic analysis was used to outline the unique spectral characteristics of the mammalian viruses within these lungs and the ecological and genetic imprints of the novel viruses. Many mammalian- or arthropod-related viruses from distinct evolutionary lineages were reported for the first time in these species, and viruses related to known pathogens were characterized for their genomic and evolutionary characteristics, host species, and locations.

**Conclusions:**

These results expand our understanding of the core viromes of rodents and insectivores from Mainland Southeast Asia and suggest that a high diversity of viruses remains to be found in rodent species of this area. These findings, combined with our previous virome data from China, increase our knowledge of the viral community in wildlife and arthropod vectors in emerging disease hotspots of East and Southeast Asia.

**Video abstract**

**Supplementary Information:**

The online version contains supplementary material available at 10.1186/s40168-020-00965-z.

## Introduction

Most human infectious viral diseases are of zoonotic origin, such as Coronavirus Disease 2019 (COVID-19) and Ebola [1–4]. There are multiple animal hosts for these zoonotic viruses, including Rodentia. Rodentia is the most diverse mammalian order and is comprised of at least 33 families and 2277 species that occur over a large variety of terrestrial habitats including both natural and human-made environments [5, 6]. Rodents live in close contact with arthropods (e.g., fleas, ticks, and mites), wildlife, and domestic animals and are a critical link in the transmission of various zoonotic pathogens between humans and animals [7, 8]. Several high-impact zoonotic viruses from the families *Arenaviridae*, *Flaviviridae Hantaviridae*, *Nairoviridae*, *Reoviridae*, and *Togaviridae* are of rodent origin [8–15]. Lassa virus (LASV), Whitewater Arroyo virus, Machupo virus, Junin virus, Guanarito virus, Tacaribe virus, and Sabia virus are examples of viruses from the family *Arenaviridae* that can cause severe human hemorrhagic fever and related diseases [16–19]. Another arenavirus (AreV) is the lymphocytic choriomeningitis virus (LCMV), which is the causative agent for human aseptic meningitis, encephalitis, and meningoencephalitis [20]. Many species of hantaviruses (HanVs), such as Hantaan virus, Seoul virus, Puumala virus, and Sin Nombre Virus, are the causative agents for hemorrhagic fever with renal syndrome (HFRS) or hantavirus pulmonary syndrome (HPS) in humans [13]. Rat hepatitis E virus (HEV) causes persistent hepatitis in humans [21]. Moreover, rodents play important roles in the natural circulation of vector-borne (especially tick-borne) viruses such as the Crimean-Congo hemorrhagic fever virus (CCHFV) of the family *Nairoviridae* and the tick-borne encephalitis virus (TBEV) and Omsk hemorrhagic fever virus (OHFV) of the family *Flaviviridae* [7, 8].

Recently, meta-genomic and meta-transcriptomic analyses have been increasingly used to survey the virome in diverse animals over a wide geographical area [22–28]. Instead of identifying the pathogens for diseases that have already emerged, virome surveillance provides abundant data to help understand the existing viral population and their ecology in ecosystems before they emerge into human populations causing large-scale outbreaks, to eventually prevent the zoonotic emerging infectious diseases (EIDs) [1, 3]. Previously, we conducted virome surveys in China that have illustrated the ecological characteristics and evolution of viruses in rodents, bats, and other small animals [29–31]. In addition to the discovery of novel viruses, many other known viruses were found in new hosts or locations that allowed a better understanding of the origin and evolutionary patterns of pathogens such as HanV, AreV, coronavirus (CoV), and arterivirus (ArteV) [10, 32–36]. These results suggest that there is still a rich as-yet-to-be-discovered viral diversity in small mammalian species and that the risk of EIDs originating from these rodents should not be underestimated.

Southeast Asia, characterized by a great diversity of mammals, a hotspot of rodent diversification, and a hotspot of biodiversity at threat coupled to cultural traditions of wildlife consumption in many regions, is identified as a hotspot for EIDs [37–41]. A number of zoonotic diseases, including viral hemorrhagic fever, severe acute respiratory syndrome, and Nipah virus-related brain or respiratory diseases, already emerged in this area [42, 43]. Accelerating environmental change and highly connected globalization, leading to increased risks of EID being shared across borders, suggests that joint efforts in surveillance, early detection, prevention, and response to EIDs are needed. For this current study, we collected 3284 lung samples from 30 rodent and insectivore (shrew, tree shrew, and short-tailed gymnure) species in 12 provinces of Thailand, three provinces of Lao PDR, and three provinces of Cambodia over a 12-year period (2006–2018) for a meta-transcriptomic analysis. The virome data obtained revealed an overview of the viral community present in the lungs of these small mammalian species across the Indochina Peninsula. The description of the host and geographical bias of these diverse viral populations and characterization of novel viruses in multiple viral communities, allowed us to identify the ecological and genetic imprints of known human and animal viral pathogens, including HanVs, phleboviruses (PhleVs), AreVs, hepaciviruses, pegiviruses, and arteriviruses (ArteVs) in these regions. This study provides a baseline that can be used for future studies to identify changes in viromes and potentially identify novel EIDs before they emerge.

## Results

### Animal sampling

**Fig. 1:**
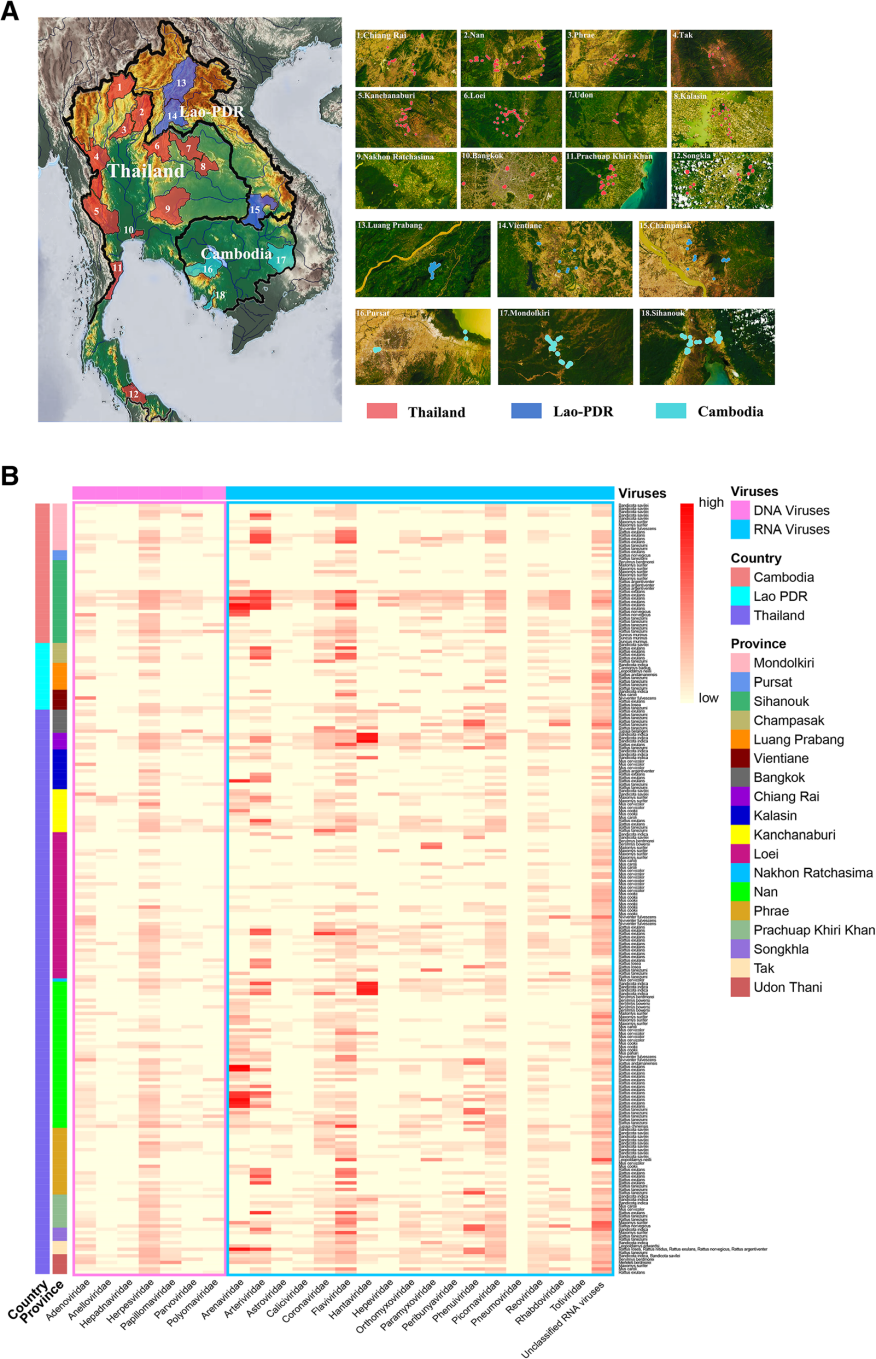
**a** Sampling sites from the various provinces of Southeast Asia. Map of Southeast Asia showing the eighteen provinces in the three countries where animals were sampled. Sampling sites are indicated in different color, with red color representing Thailand, blue representing Lao PDR, and cyan representing Cambodia. Sampling sites within the eighteen provinces are shown on the right, with dots indicating sampling sites with each province. The number of samples from the species and the provinces and dates of collection are detailed in Table S1. **b** Heatmap based on the normalized numbers of sequence reads for 24 families of mammalian viruses and a group of unclassified RNA viruses in each pooled sample. Mammalian host species are listed on the right as a text column. Location information is indicated on the left as color code, with colors defined on the right. Names of the mammalian viral families are indicated at the bottom. Relative abundance of the viruses in each species in each location are indicated as a heat map ranging from low (yellow) to high (red) based on the normalized average viral genome size and total sequencing reads in each pool

A total of 3284 lung samples of small mammal individuals were collected from different sites in the three countries between April 2006 and November 2018. These comprised 3191 lung samples from rodents, 66 lung samples from shrews and two lung samples from short-tailed gymnure under the order Eulipotyphla, and 25 lung samples from tree shrews under the order Scandentia. Samples were obtained from a total of 18 provinces, including the Bangkok urban area, Kanchanaburi, Chiang Rai, Tak, Prachuap Khiri Khan, Loei, Nan, Songkla, Udon Thani, Kalasin, Phrae, and Nakhon Ratchasima in Thailand, Vientiane, Champasak, and Luang Prabang in Lao PDR, and Preah Sihanouk, Mondulkiri, and Pursat in Cambodia (Fig. 1a). The samples included 25 species of rodents from the families Muridae, Spalacidae, and Sciuridae, three shrew species from the family Soricidae, and two species of tree shrew from the family Tupaiidae that reside in urban, rural, and wild areas throughout the Indochina Peninsula. The most commonly sampled species were *Rattus exulans*, *R. tanezumi*, *Bandicota savilei*, *B. indica*, *Maxomys surifer*, and *Mus cervicolor* (Table S1). *Rattus* was the dominant genus, accounting for 49.66% of the total samples. The genus *Bandicota* accounted for 18.30% and *Mus* accounted for 14.92% of the total samples. These rodent species are ecologically closely associated with humans, arthropod vectors, and other animals in these areas.

### Meta-transcriptomic analysis and overview of the virome

Due to the repeated sampling of some species in the same location, the 3284 isolated lung total RNA samples were combined into 233 pools with equal quantity. To determine whether viral RNA was present in each pool, an rRNA excluded RNA library was constructed and processed for NGS-based meta-transcriptomic analysis. A total of 262.38 GB of nucleotide data was obtained. Reads that were classified as cellular organisms (including bacteria, archaea, and eukaryotes) and reads with no significant homology to any amino acid sequence in the NCBI NR database were removed. The remaining 495,579 reads were used to identify their best-matched hit with viral proteins available in the NCBI NR database (Table S2). Due to the presence of numerous transcripts from the hosts and other cellular organisms, most pools had low levels of viral RNA.

To analyze the virome, virus-associated reads were classified into a group of unclassified RNA viruses and 98 known families under the double-stranded (ds) RNA viruses, reverse-transcribing viruses, single-stranded (ss) RNA viruses, dsDNA viruses, and ssDNA viruses. By further characterization of dietary habits and other host traits, non-vertebrate-associated viral reads and retrotransposon-related sequence reads that have previously been described [23, 31] were removed. The remaining 406,869 viral reads (approximately 82.1% of the total viral hits) were then assigned into 24 mammal-related viral families and a group of unclassified RNA viruses. The reads for each viral family in each pool were normalized by the viral genome size and the proportion of total viral reads, and the prevalence of each viral family in each province and animal species is shown in Fig. 1b and Table S3. Viral reads from the families *Arteriviridae*, *Arenaviridae*, *Flaviviridae*, *Hantaviridae*, *Herpesviridae*, and *Phenuiviridae* were widely distributed, in differing abundances, in a variety of rodent and insectivore species from the different regions. Virus families *Adenoviridae*, *Astroviridae*, *Anelloviridae*, *Coronaviridae*, *Caliciviridae*, *Hepeviridae*, *Herpesviridae*, *Paramyxoviridae*, *Picornaviridae*, *Peribunyaviridae*, *Rhabdoviridae*, and *Reoviridae* were found in fewer species in the different regions. Although sequence reads related to the families *Hepadnaviridae* and *Orthomyxoviridae* were occasionally present in some of the virome, when we used RT-PCR to amplify genomic sequences of these viruses, we failed to amplify any sequences. This might suggest that the *Hepadnaviridae* and *Orthomyxoviridae* viruses were of low viral load or spurious sequence similarities. In addition to the family assigned reads, a substantial number of viral reads were for unclassified RNA viruses in the realm *Riboviria*, including diverse *Chuviridae*-, *Nodaviridae*-, or *Totiviridae*-related viruses. Although most viral hits (98.64%) were assigned into the RNA virus group, a small number of DNA viral reads were found in sequence data due to their corresponding RNA transcripts being detected. Due to their low number, we did not perform any further analyses of these DNA viruses.

**Fig. 2:**
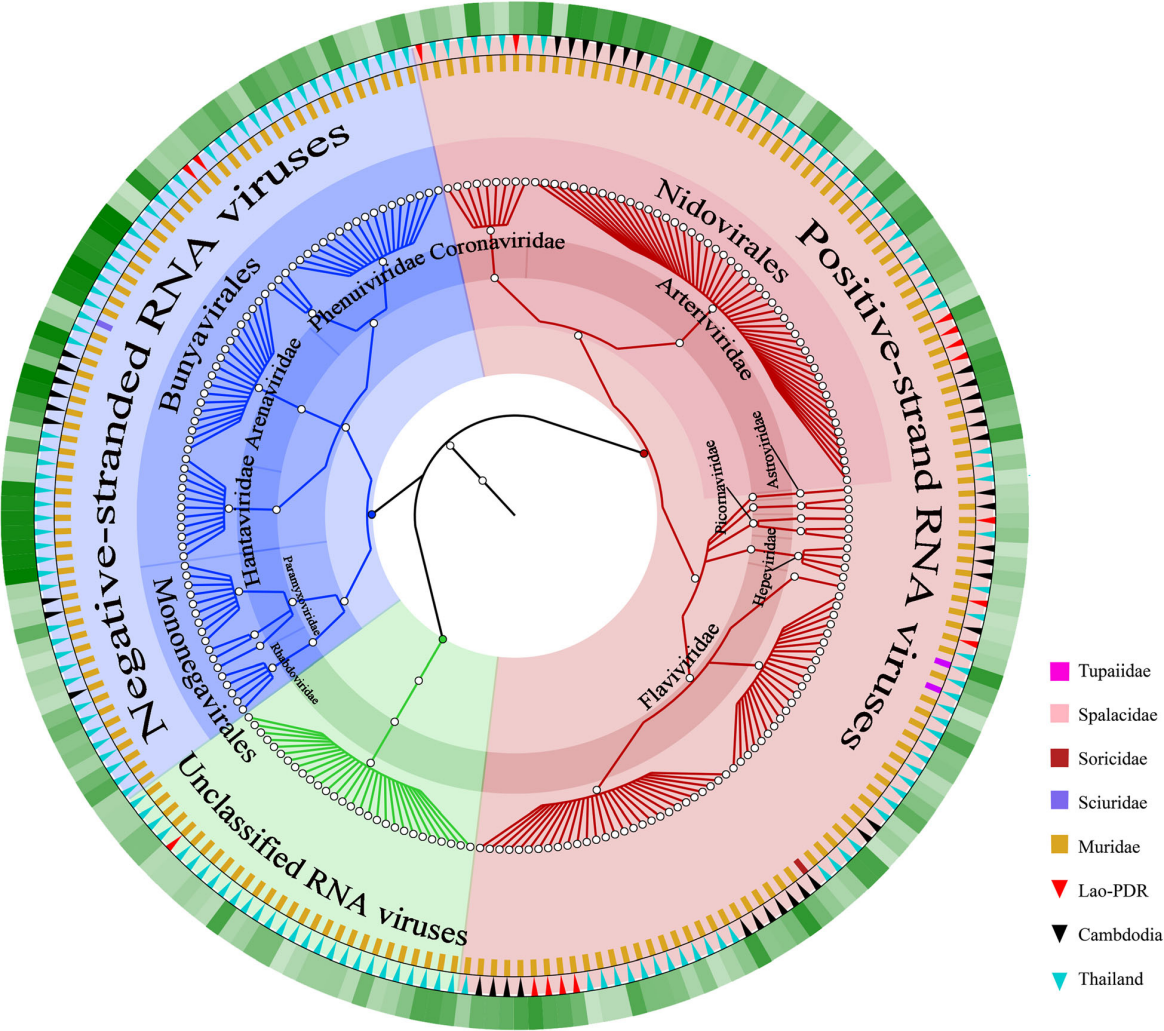
Overview of the classification, from family to order, of RNA viruses identified in this study. Different families and unclassified RNA viruses are labeled in different colors in the pie chart. The three outer rings display host mammalian family (inner ring), geographical location (middle ring), and relative abundance of virus, with light to dark green representing increasing abundance (outer ring)

Based on the virome data provided by these meta-transcriptomes, the prevalence and diversity of viruses in families including pathogens that are known to cause human and animal infection or are novel in rodent were then confirmed by PCR screening of individual lung samples. In total, 216 representative virus strains were selected for genomic or partial genomic sequencing (Fig. 2 and Table S4). Below we outline the characteristics of these different types of viruses.

### Characteristics of negative-stranded RNA viruses

#### HanVs

**Fig. 3:**
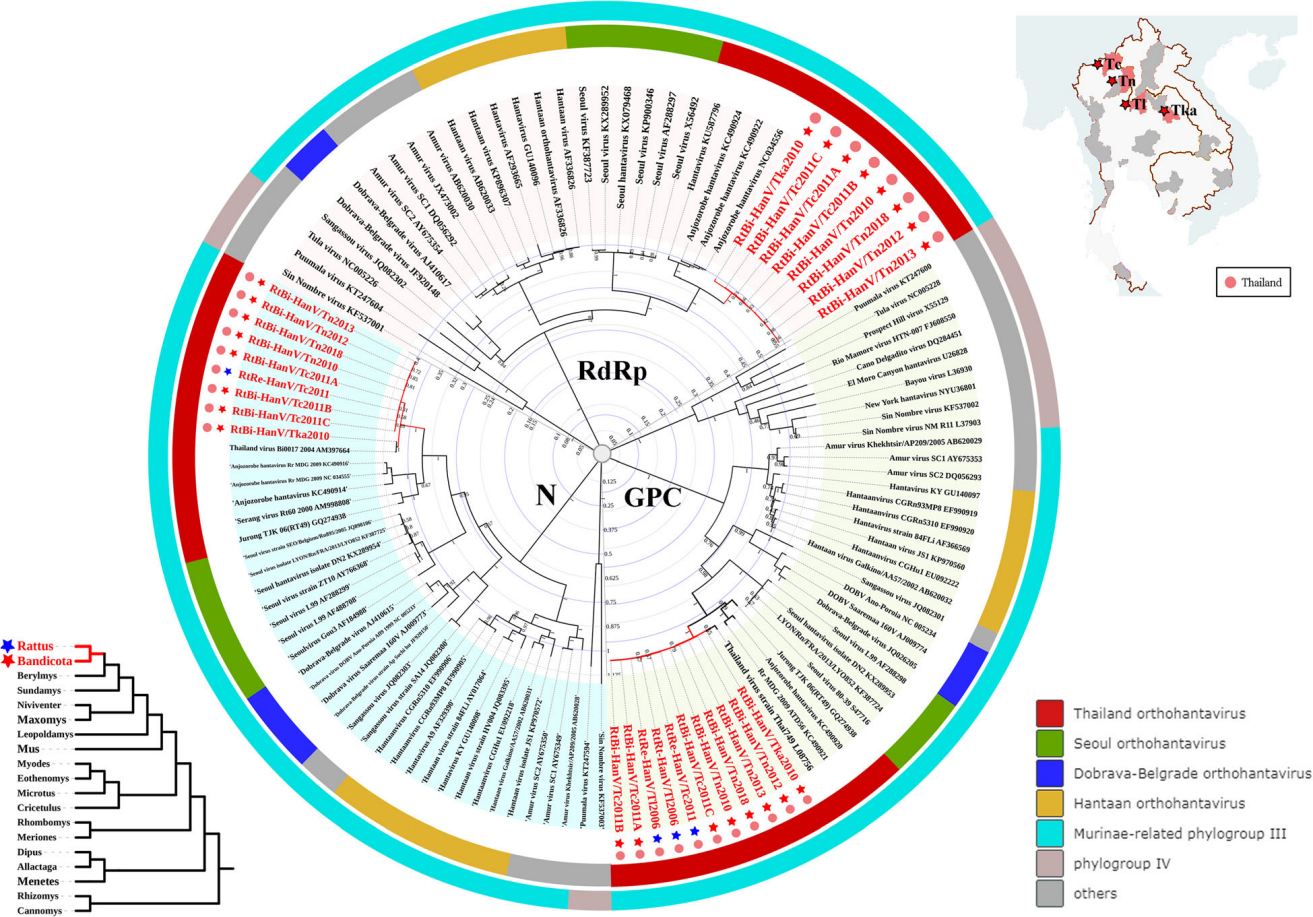
Phylogenetic trees based on the complete M segment-encoded glycoprotein precursor (GPC), L segment-encoded RdRp (RdRp), and S segment-encoded nucleocapsid protein (N) amino acid sequences of HanVs. Phylogenetic trees were constructed by the maximum likelihood method using the best-fit models (LG + G for GPC protein, LG + G + I for RdRp protein, and GTR + G + I for N protein). All HanVs found in this study are labeled in red. Host genus and location of each virus are labeled by the 5-point stars and dots of different colors. The outer color rings represent additional taxonomic information about these viruses. DOBV, Dobrava-Belgrade virus

Rodent HanVs are segmented negative-stranded RNA viruses with three genome segments (L, M, and S). These viruses of the genus *Orthohantavirus*, family *Hantaviridae* (under the order *Bunyavirales*), can be divided into two groups: the Murinae-related phylogroup III HanVs and the Sigmodontinae-, Neotominae-, and Arvicolinae-related phylogroup IV HanVs [10, 13, 44]. Here, we identified eleven HanV strains that were found in three species (*B. indica*, *R. exulans*, and *R. tanezumi*) of Murinae from five provinces (Bangkok, Chiang Rai, Kalasin, Loei, and Nan) of Thailand in multiple years (Table S4). *B. indica* is the main host for these HanVs. Eight strains were determined for genome sequences, and three strains were selected for sequencing of partial L, M, and S. The complete genome sequence (including non-coding regions (NCRs)) of these viruses showed more than 93.5% nucleotide (nt) identity with each other, suggesting that they all belonged to the same viral species. The open reading frame (ORF) for L of this species showed 79–81.4% nt identity with those of Anjozorobe HanVs detected in *R. rattus* and *Eliurus majori* from Madagascar in 2014 [45], the M and S ORFs of this species showed 95–96% and 96.1–99.4% nt identities with those of Thailand virus strains found in *B. indica* of Thailand in 1994 and 2004 [46, 47] (Table S5). Phylogenetic trees based on the complete M segment-encoded glycoprotein precursor (GPC), L segment-encoded RNA-dependent RNA polymerase (RdRp), and S segment-encoded nucleocapsid protein (N) amino acid sequences were constructed (Fig. 3). All HanVs identified here were assigned to the Murinae-related phylogroup III, clustered together, and formed a separate clade associated with the clade of Anjozorobe HanVs that are closely related to the lineage of the Seoul virus strains. Although the exact relationship between these HanVs and Thailand virus cannot be resolved, due to the absence of complete L sequences from the Thailand viruses in GenBank, we propose that a single lineage of the species *Thailand orthohantavirus* [48] includes all of the HanV strains detected in Thailand from the M, S, and partial L-based alignments and our phylogenetic analysis. This suggested that these HanVs have circulated in diverse Thai provinces for several decades.

#### PhleVs

**Fig. 4:**
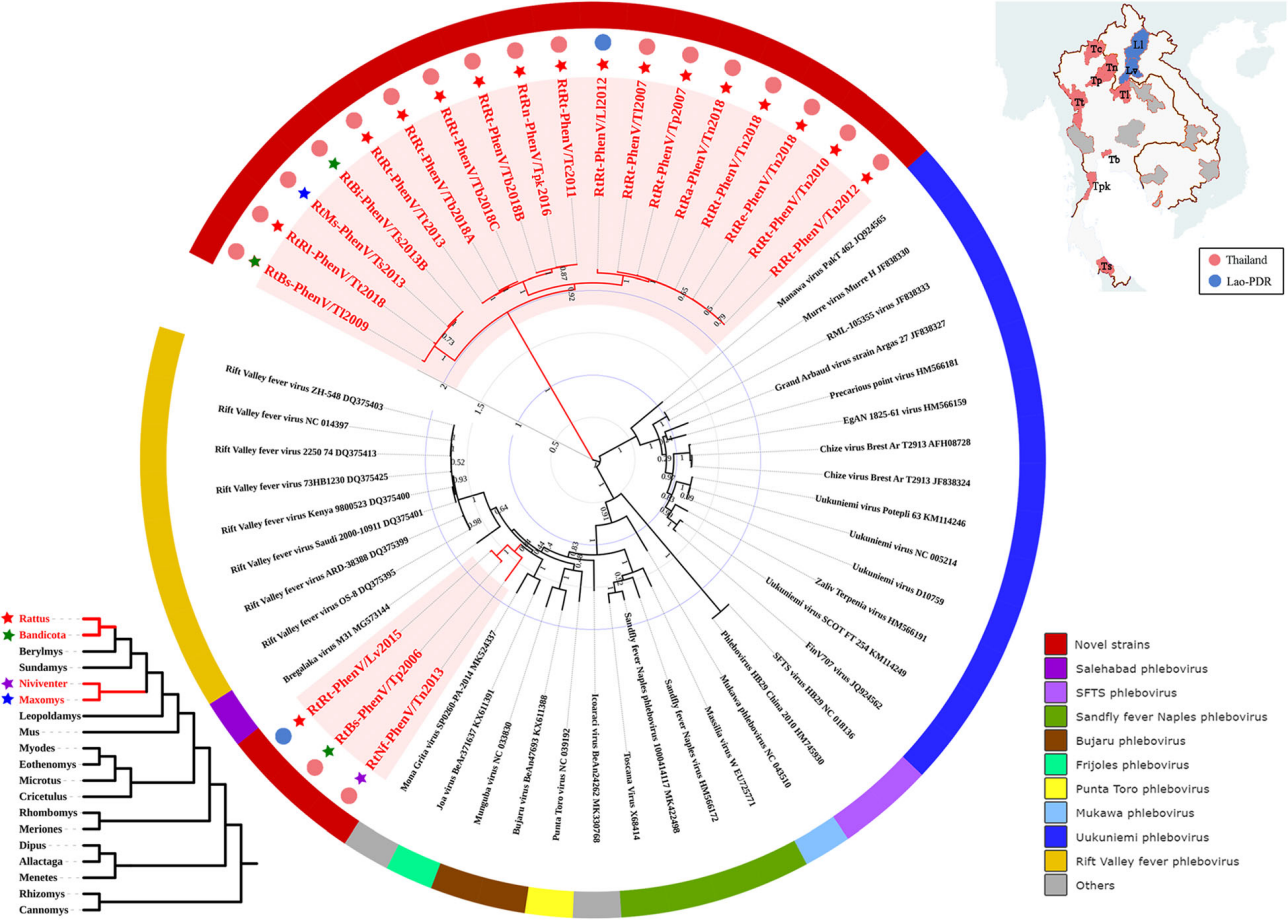
Phylogenetic tree based on the partial L (RdRP) nucleotide sequences of PhleVs (2392 bp). Phylogenetic tree was constructed by the maximum likelihood method using the best-fit model (T92 + G + I). All PhleVs found in this study are labeled in red. Host genus and location of each virus are labeled by the 5-point stars and dots of different colors. The outer color rings represent additional taxonomic information about these viruses. SFTS virus, severe fever with thrombocytopenia syndrome virus

Similar to HanVs, viruses of the genus *Phlebovirus* are also linear segmented negative-stranded RNA viruses that belong to the family *Phenuiviridae* of the order *Bunyavirales* [49]. This genus contains many highly virulent viruses such as Rift Valley fever virus (RVFV), Toscana virus, and severe fever with thrombocytopenia syndrome virus. These viruses are arthropod-borne, naturally harbored by ruminant or camel reservoirs, and are transmitted by mosquitoes, sandflies, or ticks, and cause severe diseases in humans and animals [50–52]. After mapping the sequencing reads, a total of 21 rodent PhleVs’ genome sequences were completely or partially confirmed in the lung samples from *B. savilei*, *M. surifer*, *Niviventer fulvescens*, and several diverse *Rattus* species from eight Thai provinces and two Laotian provinces (Table S4). Species of *Rattus* species were the main hosts of the PhleVs. Pairwise alignment revealed that the partially sequenced L segments of these viruses showed less than 71.2% nt identity with all other known PhleVs, which suggested that these rodent PhleVs represent novel species (Table S6). Phylogenetic analysis of the partial L nucleotide sequences revealed two distinct lineages of rodent PhleVs in the genus *Phlebovirus*, lineage 1 related to Uukuniemi PhleVs, and lineage 2 related to RVFV and Salehabad PhleVs (Fig. 4). The different PhleVs identified in species of the *Rattus* genus from different locations showed very close genetic relationships and can be further divided into two clades within lineage 1. However, some of the PhleVs from diverse rodent species shared high sequence identities and close genetic relationships. For example, RtRl-PhenV/Tt2018, RtMs-PhenV/Ts2013, and RtBs-PhenV/Tl2009 identified in three different rodent genera are closely related to each other within lineage 1, and as well, a close relationship was seen between RtBs-PhenV/Tp2006 and RtRt-PhenV/Lv2015 within lineage 2.

#### AreVs

**Fig. 5:**
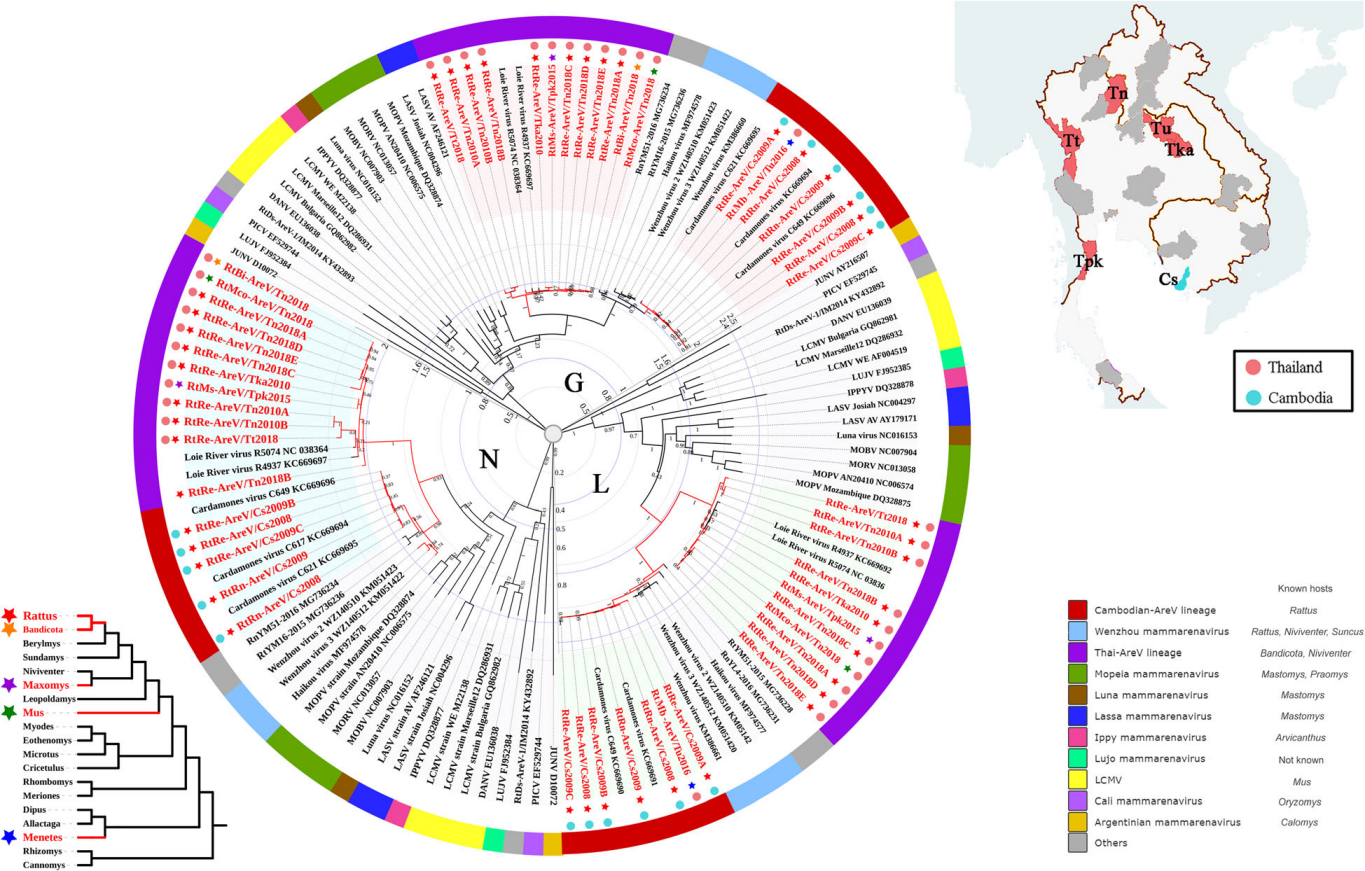
Phylogenetic trees based on the complete L protein (RdRP), glycoprotein (G), and nucleocapsid (N) protein amino acid sequences of AreVs. Phylogenetic trees were constructed by the maximum likelihood method using the best-fit models (LG + G + I for L protein, GTR + G for G protein, and T92 + G for N protein). All AreVs found in this study are labeled in red. Host genus and location of each virus are labeled by the 5-point stars and dots of different colors. The outer color rings represent additional taxonomic information about these viruses. JUNV, Junin virus; PICV, Pichinde virus; DANV, Dandenong virus; LCMV, lymphocytic choriomeningitis virus; LUJV, Lujo virus; IPPYV, Ippy virus; LASV, lassa virus; MOBV, Mobala virus; MORV, Morogoro virus; MOPV, Mopeia virus

Rodent AreVs of the genus *Mammarenavirus*, family *Arenaviridae*, can be divided into the Old-World and New-World complexes [16]. These viruses are a group of linear segmented negative-stranded RNA viruses with two genome segments (L and S). A total of nineteen Old-World AreVs strains were identified from lung samples from *B. indica*, *M. cookii*, *M. surifer*, *Menetes berdmorei*, and *Rattus* species from five Thai provinces and the Cambodian Sihanouk province (Table S4). Species of *Rattus* are the main hosts for these AreVs. Fourteen strains were characterized for genome sequences, and five strains were selected for sequencing of partial L and S. Pairwise alignment of the complete genome sequences (including NCRs) revealed that seven strains shared high sequence similarity and were closely related to Cardamones virus found in Veal Renh, Cambodia, in 2009, with 96.1–100% nt identities, and 12 strains shared high sequence similarity and were closely related to the Loei River virus found in Loei province, Thailand, in 2008, with 88.2–95.1% nt identities [53] (Table S7). In accordance with sequence alignment results, phylogenetic analyses based on the RdRP (L), glycoprotein (G), and nucleocapsid (N) proteins suggested that these AreVs could be assigned into two different lineages related to AreVs reported in China (Fig. 5). We designated them as Thai-AreV lineage and Cambodian-AreV lineage. These results revealed that the Thai-AreVs have circulated in five Thai provinces for at least 9 years (2010–2018) and that the Cambodian-AreV circulated in the Sihanouk region of Cambodian between 2008 and 2009. The only exception to this was RtMb-AreV/Tu2016, which is a member of the Cambodian-AreV lineage but was found in Udon Thani province of Thailand in 2016.

#### Rhabdoviruses (RhaVs)

**Fig. 6:**
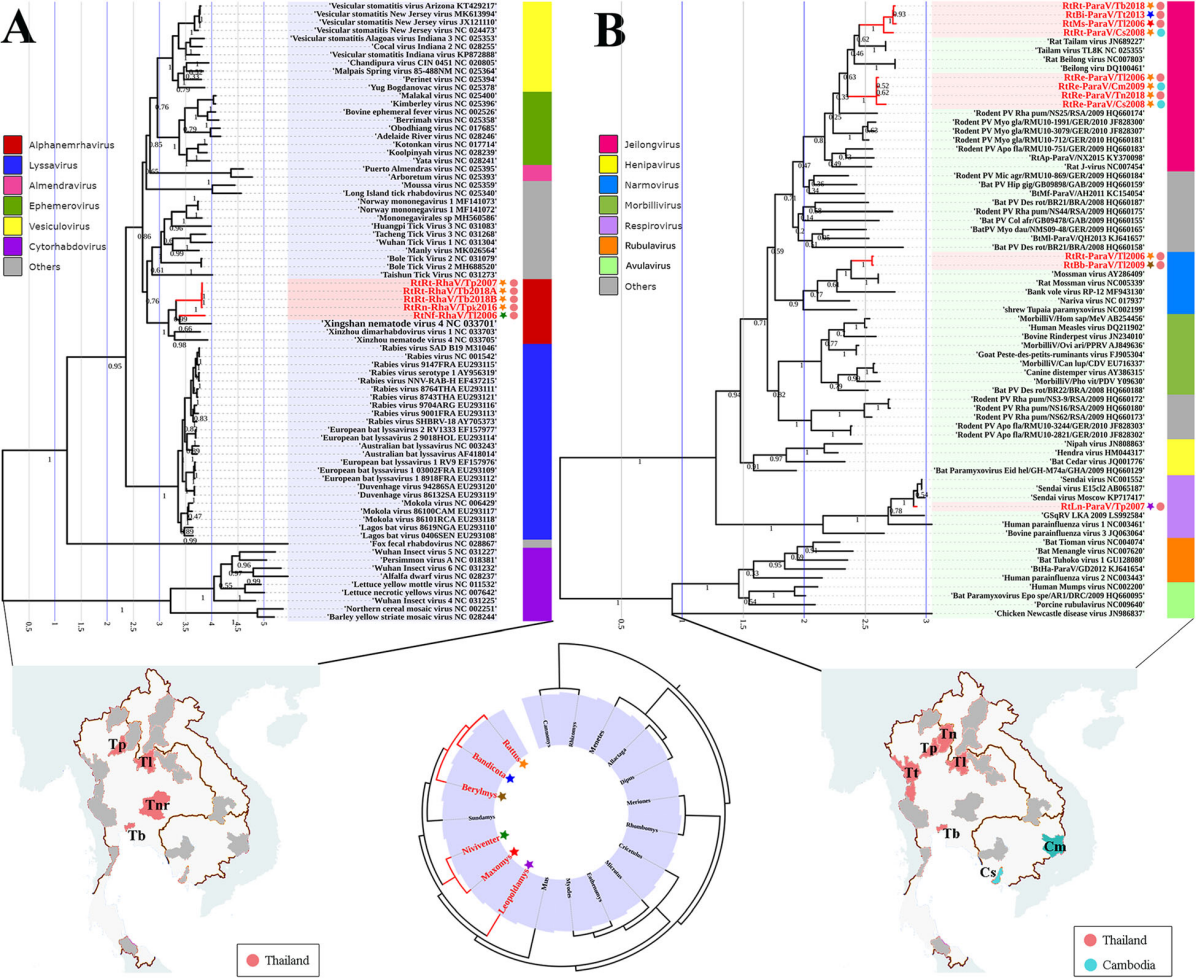
**a** Phylogenetic tree based on complete L (RdRp) nucleotide sequences of RhaV (6356 bp). **b** Phylogenetic tree based on partial L (RdRp) nucleotide sequences of ParaVs (559 bp). Phylogenetic trees were constructed by the maximum likelihood method using the best-fit models (GTR + G + I for RhaV and ParaV). All viruses found in this study are labeled in red. Host genus and location of each virus are labeled by the 5-point stars and dots of different colors. The outer color rings represent additional taxonomic information about these viruses. GSqRV, Giant squirrel respirovirus

RhaVs are a large group of linear negative-stranded RNA viruses of the family *Rhabdoviridae*, which currently has 20 genera [54, 55]. These viruses infect diverse vertebrates, invertebrates, and plants, and some of them can cause mild-to-severe diseases such as vesicular stomatitis virus and rabies virus [56, 57]. Here, we identified five RhaVs in *N. fulvescens* and *Rattus* species from four Thai provinces, and the complete genome sequences of two viruses were confirmed (Table S4). Unlike the previously reported rodent lyssavirus, mokola virus, and murine feces-associated rhabdovirus (MuFARV) [58, 59], these four RhaVs showed less than 66.5% nt homology with known *Rhabdoviridae* members (Table S8). The most closely related virus was the Xingshan nematode virus 4, a newly identified RhaV in *Spirurian nematodes* [23]. Phylogenetic analysis based on the complete L nucleotide sequences suggested that these novel rodent RhaVs clustered with the Xingshan nematode virus 4 of genus *Alphanemrhavirus* (Fig. 6a).

#### Paramyxoviruses (ParaVs)

The family *Paramyxoviridae* is a group of enveloped viruses with negative-stranded RNA genomes that are responsible for many mild-to-severe human and animal diseases [35, 60–66]. Twelve rodent ParaVs’ sequences were identified in the lung samples from *B. indica*, *Berylmys bowersi*, *Leopoldamys neilli*, *M. surifer*, and diverse species of *Rattus* from five Thai provinces and two Cambodian provinces (Table S4). We obtained full-length sequences of ten virus strains. Of these, nine ParaVs were closely related to members of the genus *Jeilongvirus* with high sequence similarity (75.3–77% nt identities for L ORF), two were Mossman virus related (75.8–76.5% nt identities for L ORF), and one was Sendai virus related (91.9% nt identities for partial sequenced L) (Table S9). Phylogenetic analysis based on the partial L nucleotide sequences revealed that these rodent ParaVs were assigned into the genera *Narmovirus*, *Jeilongvirus*, and *Respirovirus* (Fig. 6b).

### Characteristics of positive-stranded RNA viruses

#### Hepaciviruses, pegiviruses, and pestivirus

**Fig. 7:**
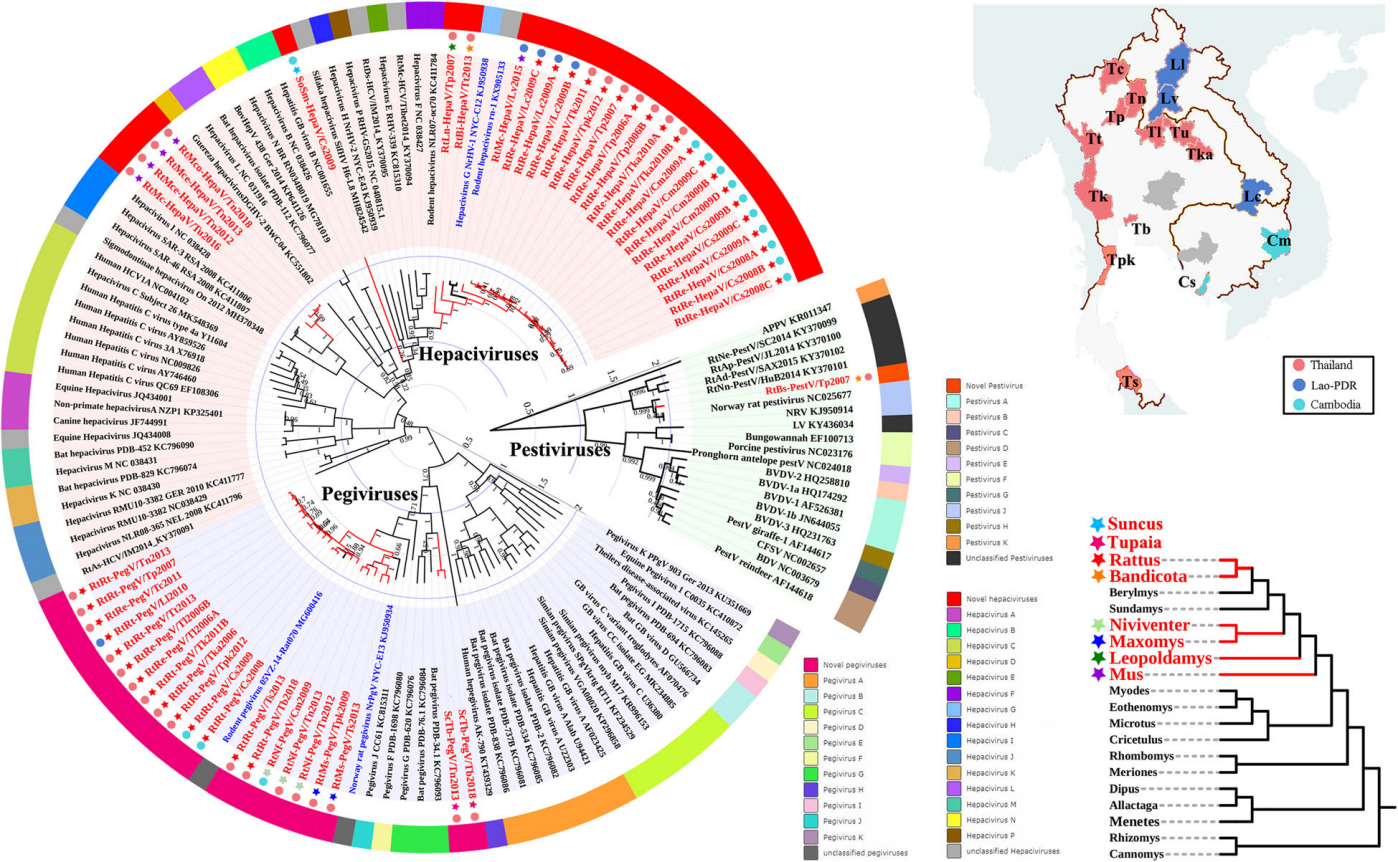
Phylogenetic trees based on the polyprotein sequences of hepaciviruses, pegiviruses, and pestivirus (hepaciviruses and pegiviruses, 2060 bp; pestivirus, 796 aa). Phylogenetic trees were constructed by the maximum likelihood method using the best-fit models (GTR + G for hepacivirus and pegivirus, rtREV+G for pestivirus). All flaviviruses found in this study are labeled in red. Host genus and location of each virus are labeled by the 5-point stars and dots of different colors. The outer color rings represent additional taxonomic information about these viruses. APPV, atypical porcine pestivirus; LV, Linda virus; BVDV, bovine viral diarrhea virus; CFSV, classical swine fever virus; BDV, border disease virus

The genera *Hepacivirus*, *Pegivirus*, and *Pestivirus* are within the family *Flaviviridae* and are positive, single-stranded RNA viruses. These viruses infect a variety of mammalian hosts, including primates, bats, horses, and rodents [67–69]. The hepatitis C virus of the genus *Hepacvirus* is an important causative agent of hepatitis and hepatocellular carcinoma in humans [70], and the two classic types of pestiviruses, bovine viral diarrhea virus and classical swine fever virus, are important causative agents of mild-to-severe disease in cattle and pigs [71, 72]. Here, we found a total of 51 viral members of the family *Flaviviridae* within the diverse rodent and shrew species lung samples from almost all sampling sites across Thailand, Lao PDR, and Cambodia (Table S4). Thirty-four strains underwent genome sequencing and seventeen strains were selected for sequencing of partial polyproteins. Pairwise alignment of the complete or partial genome sequences suggested that twenty-eight of them were hepaciviruses, with 41.4–100% nt identities with each other, twenty-two were pegiviruses, with 42.1–96.1%% nt identities with each other, and one was pestivirus, with 75.2% nt identity to a known rodent pestivirus (Table S10). Phylogenetic analysis based on the partial polyproteins revealed that the 51 novel viruses could be assigned to distinct novel lineages within the genera *Hepacivirus*, *Pegivirus*, and *Pestivirus* (Fig. 7). Several host-specific lineages, including a *Rattus exulans*-related lineage for *Hepacivirus*, and *N. fulvescens*-related and *Rattus*-related lineages for *Pegivirus*, were detected that suggested that the phylogenies of most of these viruses were strictly congruent with the relationships of their rodent or insectivore hosts. For the first time, we reported hepacivirus and pegivirus (SoSm-HepaV/Cs2009, ScTb -PegV/Tb2018, and ScTb-PegV/Tn2013) for insectivores (shrew and tree shrew). The virus SoSm-HepaV /Cs2009 represented a separate hepacivirus clade with less than 44.4% nt identity with any known virus. ScTb -PegV/Tb2018 and ScTb-PegV/Tn2013 represent a separate pegivirus clade with less than 57.6% nt identity with known viruses. These viruses, together with previously reported bat viruses, formed the main evolutionary frames for these two genera.

#### ArteVs

**Fig. 8:**
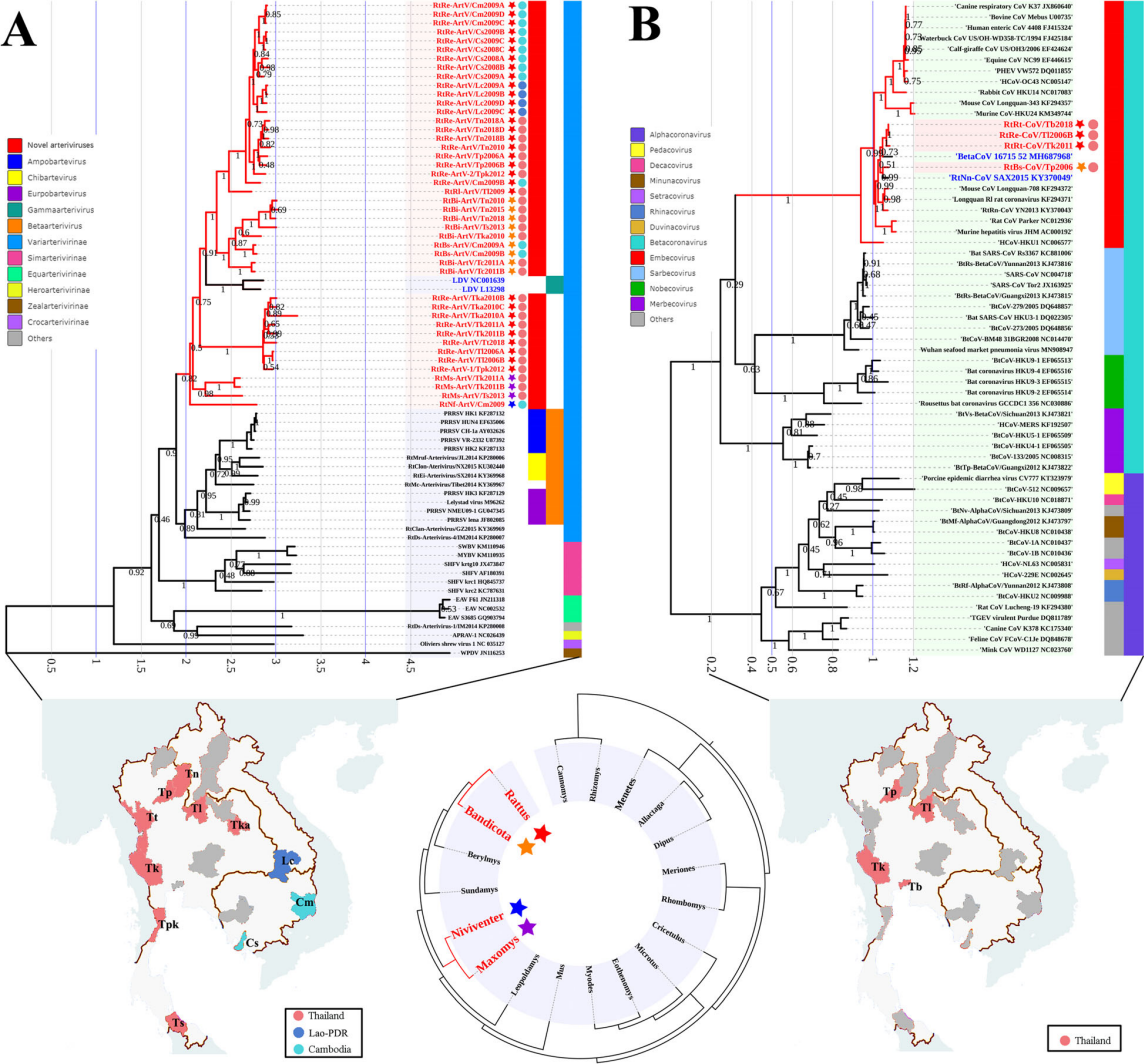
**a** Phylogenetic tree based on complete ORF1b nucleotide sequences of ArteVs. **b** Phylogenetic tree based on complete RdRp nucleotide sequences of CoVs. Phylogenetic trees were constructed by the maximum likelihood method using the best-fit models (GTR + G for ArteV, and GTR + G + I for CoV). All viruses found in this study are labeled in red. Host genus and location of each virus are labeled by the 5-point stars and dots of different colors. The outer color rings represent additional taxonomic information about these viruses. SWBV, southwest baboon virus; MYBV, Mikumi yellow baboon virus; APRAV, African pouched rat arterivirus; WPDV, Wobbly possum disease virus

ArteVs, of the family *Arteriviridae*, are a group of enveloped viruses with positive single-stranded RNA genomes that are responsible for a variety of mild to severe diseases in horses, simians, and swine, such as equine arteritis virus (EAV), simian hemorrhagic fever virus (SHFV), and porcine reproductive and respiratory syndrome virus (PRRSV) [73–76]. A total of 49 ArteVs’ genomic or partial genomic sequences were identified in diverse rodent species from almost all sampling sites throughout Thailand, Lao PDR, and Cambodia (Table S4). Pairwise alignment of ORF1b revealed that these viruses shared 55.7–98.6% nt identities with each other, and less than 66% nt identity with known ArteVs (Table S11). These sequences are most similar to previously reported *Betaarterivirus* and *Gammaarterivirus* members of the genera, PRRSVs, lactate dehydrogenase-elevating virus (LDV), and unclassified rodent ArteVs. However, unlike the results of our previous study of rodent pharyngeal and anal samples from China that showed a diverse phylogenetic scattering of ArteVs throughout the family *Arteriviridae*, here, phylogenetic analysis based on ORF1b and ORF5 revealed that all ArteVs found here in lung tissues clustered with each other as a separate clade within the subfamily *Variarterivirinae* with different host-specific lineages (such as *Rattus*-related, *Maxomys*-related, and *Bandicota*-related lineages) (Fig. 8a). These lineages represented distinct viral classifications that differ from the previously identified genera *Betaarterivirus* and *Gammaarterivirus* of the subfamily *Variarterivirinae*.

#### CoVs

CoVs are a group of enveloped viruses with a large positive single-stranded RNA genome within the subfamily *Coronavirinae* and include viruses that result in human diseases such as colds, severe acute respiratory syndrome (SARS), Middle East respiratory syndrome (MERS), and COVID-19 [1, 3, 77]. The subfamily *Coronavirinae* is divided into four recognized genera, *Alphacoronavirus*, *Betacoronavirus*, *Deltacoronavirus*, and *Gammacoronavirus* [78–81]. Previously, we had found a large number of rodent CoVs in the diverse rodent species in China that were assigned into two separate lineages within *Alphacoronavirus* and *Betacoronavirus* [29]. Here, however, we only found nine CoVs within lung samples from species of *Bandicota* and *Rattus* from five Thai provinces and the Laotian Champasak province, and two of them were identified for genome sequencing (Table S4). Sequence similarity and phylogenetic analysis of RdRp revealed that all of these CoVs could be classified within *Embecovirus* under the genus *Betacoronavirus*, with nt sequence identities between 93.7 and 100% (Fig. 8b and Table S12). Despite our large sample size, alpha-CoV was not found in our samples.

#### Hepatitis E viruses (HEVs)

**Fig. 9:**
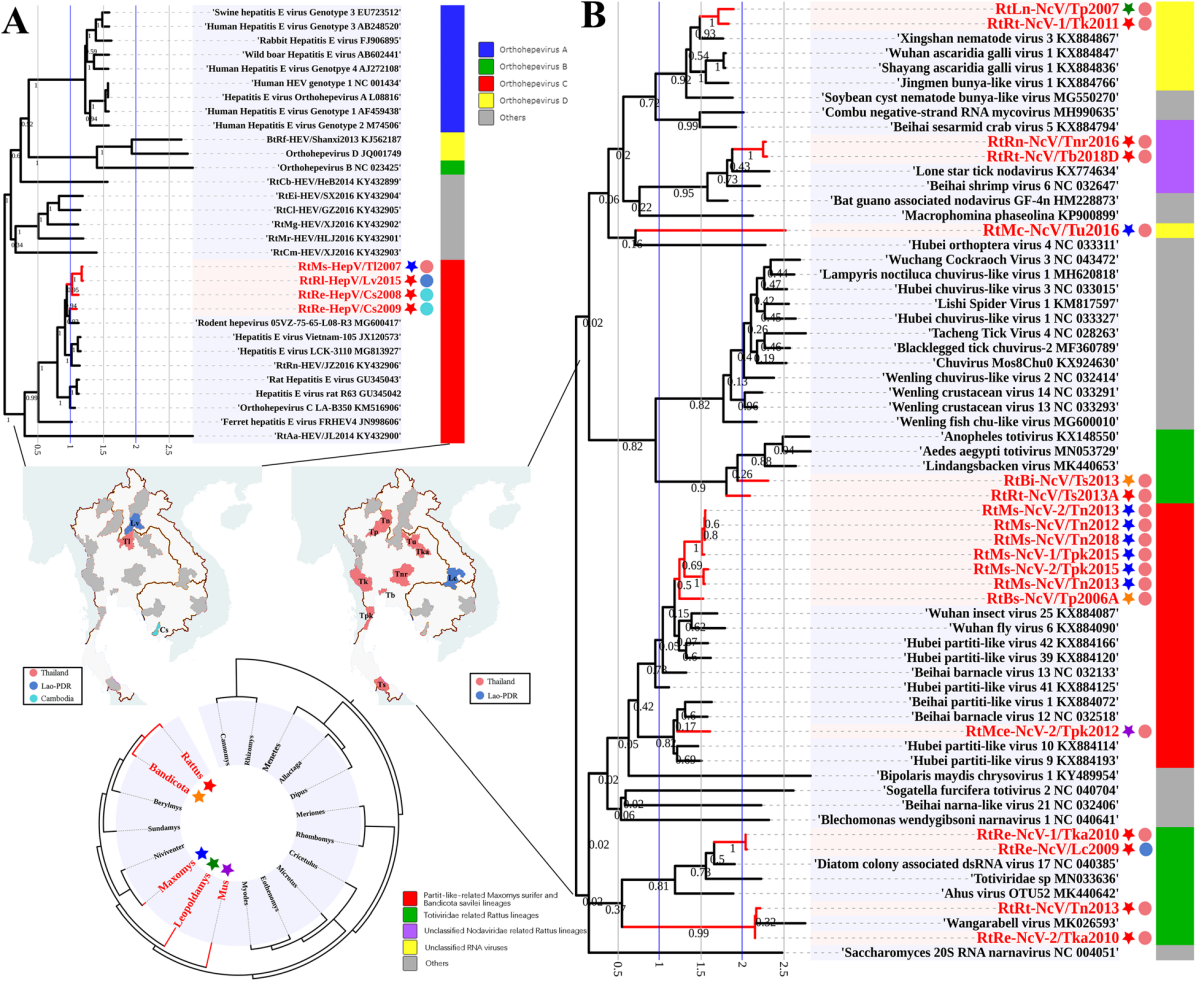
**a** Phylogenetic tree based on partial ORF1 nucleotide sequences of HEVs (4392 bp). **b** Phylogenetic tree based on partial RdRp nucleotide sequences of unclassified RNA viruses (1014 bp). Phylogenetic trees were constructed by the maximum likelihood method using the best-fit models (GTR + G for HEV, and GTR + G for unclassified RNA viruses). All viruses found in this study are labeled in red. Host genus and location of each virus are labeled by the 5-point stars and dots of different colors. The outer color rings represent additional taxonomic information about these viruses

HEVs of the family *Hepeviridae* are a group of small, non-enveloped, positive single-stranded RNA viruses. Members of the species *Orthohepevirus A* are one of the most common causative agents of hepatitis in humans, and rodent-borne *Orthohepevirus* C was recently reported to be zoonotic and cause persistent hepatitis in humans [21, 27, 29, 42, 82]. The partial genome sequences of four HEVs were identified in *M. surifer* of Thai Loei province, *R. losea* of Laotian Vientiane province, and *R. exulans* of Cambodian Sihanouk province (Table S4). All of these viruses were closely related to previously reported rodent HEV, strains Vietnam-105, and human patient HEV strain LCK-3110, with 77.7–80.7% nt identities in ORF1, but less than 58.9% nt identity in ORF1 compared to HEVs from other hosts (Table S13). Phylogenetic analysis based on ORF1 assigned these HEVs into the species *Orthohepevirus* C, and they were closely related to the Vietnam-105 and LCK-3110 lineage, which was suspected to be a causative agent of human persistent hepatitis (Fig. 9a).

#### Picornaviruses (PicoVs)

Members of the family *Picornaviridae* are small, non-enveloped, positive single-stranded RNA viruses. Diverse PicoVs cause mucocutaneous, encephalic, cardiac, hepatic, neurological, and respiratory diseases in a wide variety of vertebrate hosts [83]. In our samples, three PicoVs were identified in *B. indica* of Thai Chiang Rai province, *R. tanezumi* of Laotian Luang Prabang province, and *R. tanezumi* of Cambodian Sihanouk province (Table S4). Sequence similarity and phylogenetic analysis of complete RdRp indicated that all of these PicoVs were closely related to known rodent PicoVs with nt sequence identities between 42 and 88.4% (Table S14 and Figure S10).

#### Astrovirus (AstroV)

AstroVs comprise positive single-stranded RNA viruses and are members of the genus *Mamastrovirus* within the family *Astroviridae* and infect many mammals and cause gastroenteritis [84]. We identified only one AstroV in *B. savilei* of the Thai Loei province. This virus shared 82.05 % nt identity in complete RdRp with an AstroV previously reported in China (Table S16 and Figure S11).

### Characteristics of unclassified RNA viruses

Recent viral surveillance studies in invertebrates, amphibians, reptiles, and fishes have revealed a new view of the RNA virosphere that is more diverse than the current taxonomy [23, 26]. Here, we identified a total of 28 unclassified RNA viruses that were found in the lungs of diverse rodent species from different Thai and Laotian provinces (Table S4). After ORF annotation for these complete or partial sequenced viruses, pairwise alignment revealed that the code regions of partial RdRp for these viruses showed 22.7–99.9% nt identities with each other, and less than 66.7% nt identity with other known RNA viruses (Table S16), suggesting that these newly identified viruses are highly diverse and distinct from known and undefined viral families. To further examine the evolutionary relationships between these viruses, phylogenetic trees were constructed based on the partial RdRp proteins of viral genomes from all related known families, genera, and unclassified taxa of invertebrates, amphibians, reptiles, and fish. The unclassified RNA viruses found here formed at least 10 distinct lineages (Fig. 9b). Most of the viruses tended to form different lineages such as the partit-like-related viruses found in the *M. surifer* and *B. savilei* lineages, the *Rhabdoviridae*-related viruses found in the *Rattus* lineage, and the *Totiviridae-*related viruses found in the *Rattus* lineage. These data suggested that RNA viruses in rodents occupy a broader range of phylogenetic diversity and are similar to the RNA viral spectrum observed in invertebrates.

## Discussion

As many animal-borne pathogens are transmitted through the respiratory tract, oral cavity, or enteric canal, previous studies have focused on systemically deciphering the viral communities found in respiratory or fecal samples (which are easy to get through non-invasive sampling) from natural animal hosts [25, 29–31, 58]. But identification of novel viruses found in open environmental samples raises question of whether these viruses are harbored by the animal host and cause actual infections, or they are just viruses that infect insect, fungi, and plants (and other environmental sources) associated with the life habits of this animal host. Although our previous reports provided evolutionary evidence for the zoonotic origin of viruses such as henipavirus, HanV, AreV, SARS-CoV, and MERS-CoV [10, 32–36, 85, 86], the presence of numerous vertebrate-irrelevant viral sequences in oral and rectal virome data complicated the definition of the host-virus relationships. Hence, organ- or tissue-based virome analysis, designated as “core virome” analysis, is also needed to accurately describe the viral infection status in natural reservoirs. This effort should give us a better understanding of which threats are directly coming from the infections of natural hosts and which threats just originate from the environment. Recently, a meta-transcriptomic-based virome analysis of the internal organs of amphibians, reptiles, and fish conducted by Shi et al identified extremely distant vertebrate-associated viruses and defined the long evolutionary history of vertebrate RNA viruses [26]. However, to date, large-scale core virome surveillance has not been performed to understand the ecological diversity of the viral community in the internal organs or tissues of rodents, the largest mammalian group and one of the most important natural reservoirs of many zoonotic viruses with severe impacts on human populations [7].

For the first time, this study details the core viromes residing in the lungs of rodents and other small insectivores throughout the three main countries of Mainland Southeast Asia. Muridae and Spalacidae rodents represent a large part of the rodent diversity in Mainland Southeast Asia and were the main focus of this study. With 10 genera and 24 species from the families Muridae and Spalacidae represented in our samples, our study includes most rodent species living in human-modified and natural habitats in the region [87–89]. Sampling of species belonging to the families Sciuridae, Erinaceidae, Soricidae, and Tupaiidae was opportunistic and represent only a small fraction of the total taxonomic diversity within these families. The lung was used for this meta-transcriptomic analysis as the lung is the organ where many viral infections happen in animals, as well as the place where viral spillover initiates between species [7, 8]. By greatly reducing the interference by irrelevant environmental microorganism, the actual abundances of RNA viruses and the transcriptional levels of DNA viruses in the lung organs was revealed. We identified new viral families, genera and species with the characterization of 216 diverse unclassified RNA viruses, HanVs, PhleVs, AreVs, RhaVs, ParaVs, hepaciviruses, pegiviruses, ArteVs, CoVs, HEVs, and PicoVs, extending our knowledge of the viral biodiversity of rodents in the Indochinese Peninsula, albeit the roles of some of these viruses in disease remain unclear. The characterization of a number of the unclassified RNA viruses that are different from all known viral families suggests that our knowledge of rodent viral richness is still very limited and that there is an important number of “missing viruses” needing further investigation.

The core virome harbored by the rodent internal organs is quite different from the virome found in nasopharyngeal or rectal swabs previously [24, 29, 58, 85, 86]. The widely distributed RNA viruses, such as various PheVs, hepaciviruses, pegiviruses, and ArteVs detected in this study, were not or only occasionally found in the oral and anal samples previously described. In contrast, RNA viruses that mainly infect their hosts through the upper respiratory tract or enteric canal, such as pestivirus, PicoV, HEV, AstroV, sapovirus, norovirus, rotavirus, morbillivirus, and picobirnavirus, were seldomly or never detected in the internal organ samples screened in this study [27, 28, 66]. This difference may due to the different tissue tropisms of these viruses: viruses such as PheVs, hepaciviruses, and pegiviruses are blood- or body fluid-borne pathogens while viruses such as pestivirus, PicoV, HEV, and morbillivirus are fecal-oral or respiratory-transmitted pathogens. The blood- or body fluid-borne viruses could be detected in various internal organs with different viral loads because of their different infection cycles, viremia, or inevitable cross-contamination of tissue and blood during sampling, while the fecal-oral or respiratory-transmitted viruses could also been occasionally detected in internal organs because of their diverse replication features or viremia. Compared to the widely distributed beta-CoVs identified in rodent oral and rectal samples, only a few beta-CoVs were detected in the lung specimens, suggesting that beta-CoV infection mainly occurs in the upper respiratory tract of rodents. Furthermore, the absence of rodent alpha-CoVs in any of the lung sample suggests that this CoV group may not be responsible for lower respiratory tract infection in these rodent species. Similarly, all lung ArteVs show a separate evolutionary footprint differing from the previously reported PRRSV-related rodent ArteVs identified in the oral and anal specimens, also suggesting two different types of ArteV infection in rodents.

By showing that species of small mammals that live in the Indochinese Peninsula carry greater numbers of viruses than previously thought, we extend the known host and geographical ranges of many viral families. Most of the viruses identified in this study show no strict geographical or host restrictions. Here, we first report some viruses in specific hosts, such as shrew hepacivirus and pegivirus, tree shrew pegivirus, and a large group of novel rodent PheVs, which indicates that these species may harbor a greater diversity of both known and unknown viruses, some of which may have zoonotic potential. A large number of viruses were identified in rodents belonging to the genera *Rattus* and *Bandicota*. This suggests that these two genera might act as major reservoirs for diverse mammalian viruses throughout the Mainland of Southeast Asia. Species members of these two genera are characterized by their synanthropic behavior and have been implicated in the emergence and spread of infectious diseases of public health importance, such as plague, murine typhus, scrub typhus, and leptospirosis, in addition to diseases caused by viruses [90].

HanV and AreV of rodent origin are important causative agents of human diseases. Almost all previously reported phylogroup III HanVs and Old-World AreVs are Murinae borne, with the exceptions of Jerboa hantavirus and Jerboa arenavirus we identified recently [91]. The Thailand virus from *B. indica* is the only reported hantavirus species carried by rodents in Thailand [46, 47]. Although this virus is suspected to be the causative agent of HFRS, only a few fragmentary pieces of sequence information for this virus are available so far. We identified Thailand virus-related HanVs in *B. indica* and two *Rattus* species in five Thai provinces between 2006 and 2018, indicating that this viral species of phylogroup III HanV is continuously circulating in different Murinae species in Thailand. The first confirmed genomic sequences of this virus obtained in this study enable us to further understand the phylogenetic relationship between the Thailand virus and other HanVs. Similarly, the identification of Thai- and Cambodian-AreV lineages here reveals the continual existence of AreVs in Indochinese Peninsula, and the relatively strict geographical restrictions of AreVs in this region. However, we found a Cambodian-AreV, RtMb-AreV/Tu2016, in *M. berdmorei* of the Thai Udon Thani province, which is geographically distant from the Cambodian Sihanouk province, suggesting that this virus was disseminated recently to additional host species over a wider geographical range. And furthermore, the identification of RtMb-AreV/Tu2016 in *Menetes* species of the family Sciuridae indicate again that Old-World AreV are able to infect more mammalian hosts than previously thought and that Murinae species are not their only hosts. Since this AreV is confirmed to be involved in human infections in Southeastern Asia [53], the potential for disease emergence from rodent-origin AreV in this region should not be underestimated.

Rodents can serve as potential sources for vector-borne disease outbreaks and many arboviruses such as CCHFV of the family *Nairoviridae*, TBEV and OHFV of the family *Flaviviridae*, and Venezuelan equine encephalitis virus of the family *Togaviridae* are harbored by rodents and are transmitted to humans and other animals via vectors such as the tick, mosquito, or sand-fly [8]. Here, we found diverse rodent viruses in the lung that are related to arthropod viruses, including the families *Phenuiviridae*, *Rhabdoviridae*, *Totiviridae*, and *Chuviridae*, and a large group of unclassified RNA viruses. These viruses show both close and distant phylogenetic relationships with known arthropod viruses, suggesting that rodents may act as one of the mammal reservoirs for these arthropod-related viruses in the environment, and play roles in the arthropod-cycle and the conservation of such viruses. Since Mainland Southeast Asia is a hotspot for many arbovirus-related tropical diseases [42, 43], the threat from rodent reservoirs should not be ignored.

## Conclusions

These findings, combined with our previous viral surveys from rodents, bats, and mosquitoes, and the online viral databases for these animals (DBatVir, DRodVir, and DMosVir, http://www.mgc.ac.cn/) [29–31, 92–94], greatly increase our knowledge of the viral community in wildlife and arthropod vectors in densely populated countries of East and Southeast Asia. Continued efforts in viral surveillance among wildlife hosts will reveal greater diversity of viral lineages, as recently hypothesized globally [37] and provide evidence to mitigate the risk of potential zoonotic disease emergence, as well as build the regional and global capacity for effective prevention and response to EIDs [22].

## Methods

### Animal samples

Rodents were trapped once or twice (in the wet season between June and October, and in the dry season between December and March) over a 12-year period between 2006 to 2018. Trapping sessions were conducted once or twice annually for each locality, with the trapping conducted over a 4-night period, with 30 lines of 10 traps placed in three different habitats, namely, (1) forest and mature plantations, (2) non-flooded lands or fields (shrubby waste land, young plantations, orchards), and (3) rain-fed lowland paddy rice fields (cultivated floodplain) (with the exception of Bangkok Metropolitan recreational parks). This yielded a total of 1200 night-traps per trapping session. Locally made live cage traps were used. At each locality, the 30 lines were placed within a 10-km^2^ area. Each line was separated from others by at least 1 km (different habitats) or 3 km (same habitat). Villages and isolated houses, which correspond to a fourth habitat category, human settlement, were also sampled opportunistically using cage traps distributed to residents [43]. The locations of sampling sites were recorded by place name and GPS coordinates with latitude and longitude. The identity of the captured species was initially determined by morphology by experienced field biologists and subsequently confirmed by barcoding of mitochondrial cytochrome b, mitochondrial cytochrome oxidase I, or 16sRNA sequences [40, 95]. Complete data on the animals used as reference specimens for the barcoding assignment is available on the “Barcoding Tool/RodentSEA” section of the CERoPath project website (www.ceropath.org). Captured animals were humanely euthanized and lung organ was collected. Each lung sample was placed in a cryogenic vial and temporarily stored in liquid nitrogen before being transported to the laboratory and stored at − 80 °C.

### RNA library construction and next-generation sequencing

Lung samples were washed with Hank’s balanced salt solution twice and then homogenized using a TissueLyser (Qiagen) in the presence of lysis buffer (Buffer RLT Plus) and stainless-steel beads (5 mm mean diameter). Total RNA was extracted from each sample using a RNeasy Plus Mini Kit (Qiagen) and quantified by a Qubit Fluorometer (Thermo Fisher Scientific). For library construction, RNAs from the same or related rodent species and collected at the same or related sites were pooled in equal quantity and then checked for integrity by an Agilent 2100 Bioanalyzer (Agilent Technologies), rRNA was removed from each pool using NEBNext rRNA Depletion Kit (NEB), and the final libraries were prepared using the NEBNext Ultra II RNA Library Prep Kit for Illumina (NEB). For sequencing, each pool was labeled with a specific index using NEBNext Multiplex Oligos for Illumina (NEB) and then processed for next-generation sequencing (NGS) using an Illumina HiSeq2500 sequencer (Illumina) using the 125 bp paired-end method. Raw sequence reads were filtered using previously described criteria to obtain valid sequences, and reads with no call sites, sequencing adaptor reads, duplicate reads, and low-complexity reads were removed [31, 96].

### Taxonomic assignment and viral RNA quantification

Sequence similarity-based taxonomic assignment was conducted as previously described [31]. Briefly, sequence reads were evaluated for viral origin by conducting alignments with the NCBI protein database (NR), using BLASTx (-e 1e-5 –F T). To detect potential viruses with remote similarities, reads with no hits in the NCBI NR database were further assembled using the software Trinity (v.2.4.0), and the resulting contigs were aligned again to NCBI NR to identify any viral-like sequences. The taxonomic identity of the aligned reads and contigs with the best BLAST scores (E-value < 10^−5^) were then parsed and exported using a MEGAN 6 - MetaGenome Analyzer. To estimate the abundance of the viral RNAs in each pool, reads were assigned to each viral taxon at the level family and were normalized by the total number of sequencing reads (or using previously described VTMK-index [96]) and viral genome size. While maintaining a low false-positive rate, any viral taxon supported by less than three unique reads was excluded from the quantitative analysis.

### Calculation of viral prevalence and the full-genome sequencing of each virus in the positive samples

Sequencing reads and assembled contigs for the same family or genus of each virus were extracted from MEGAN 6 - MetaGenome Analyzer. The accurate locations of these reads and contigs and the relative distances between them were determined using the alignment results from BLASTx or BLASTn. A draft genome, which contained the single nucleotide polymorphisms from each virus, was obtained. Specific primers were designed from the located sequence reads and contigs to screen for each virus in the individual samples from each mammalian species. Representative positive samples for each virus were selected for genome sequencing as a viral quasi-species. Fragments between the reads and contigs were amplified with overlapping, nested specific primers, and then sequenced. The majority of the viral genomes were obtained by merging sequence reads, assembled contigs, and amplified fragments using the SeqMan program (Lasergene). Any remaining gaps and the termini of genomes were determined using genome walking, inverse PCR, and 5'- and 3'-rapid amplification of cDNA ends.

### Genome annotation

The ORFs in the complete and partial genomes of the sequenced viruses were predicted with the NCBI ORF Finder tool (https://www.ncbi.nlm.nih.gov/orffinder/). Changes in the nucleotide sequences of the genomes and the amino acid sequences of ORFs were deduced by comparing the sequences with those of related viruses available in GenBank. Conserved protein families and domains were predicted using InterProScan 5 (available at: http://www.ebi.ac.uk/services/proteins). Pairwise and multiple sequence alignments were performed by using Clustal Omega, Needle (http://www.ebi.ac.uk/Tools/), MegAlign (Lasergene), and T-coffee (http://tcoffee.crg.cat/apps/tcoffee/index.html) with manual curation.

### Phylogenetic analysis

All related reference viral sequences were downloaded from GenBank. We used MEGA6.0 to align the nucleotide and deduced amino acid sequences, using the MUSCLE program with default parameters [97]. The best substitution model was then evaluated with the Model Selection package. We constructed a maximum likelihood phylogenetic tree in MEGA6.0, using the appropriate substitution model, with 1000 bootstrap replicates. Phylogenetic trees were displayed, manipulated, and annotated using The Interactive Tree Of Life (https://itol.embl.de) [98].

### Data accessibility

All genome sequences were submitted to GenBank. Accession numbers for the viruses are MT085080 to MT085341 (Table S4). Illumina HiSeq2500 sequence data was deposited into NCBI BioProject under accession number PRJNA605875.

### Description of Supplementary Information (SI)

Supplementary Tables and Figures are available with the online version of this paper.

## Supplementary Information

**Additional file 1. Table S1.** Samples of the 30 animal species used in this study and the countries (provinces) and dates of collection. **Table S2.** Overview of virus-associated reads. **Table S3.** Overview of sequence reads from mammal related viral families and unclassified RNA viruses. **Table S4.** Origin and accession number of viruses identified in this study. **Table S5.** Nucleotide sequence identity of novel rodent hantaviruses and known hantaviruses within the L, M, and S segment. **Table S6.** Nucleotide sequence identity of novel rodent PhleVs and known PhleVs within the L open reading frame. **Table S7.** Nucleotide sequence identity of novel rodent arenaviruses and known arenaviruses (AreV) in the L-, G- and N-encoding regions. **Table S8.** Nucleotide sequence identity of novel rodent rhabdoviruses and known rhabdoviruses (RhaV) in the L protein-encoding regions. Table S9. Nucleotide sequence identity of novel rodent paramyxoviruses and known paramyxoviruses (ParaV) in the L protein-encoding regions. Table S10. Nucleotide sequence identity of novel rodent and known hepaci-, pegi- and pestviruses. Table S11. ORF1b nucleotide sequence identity of novel rodent and known arteriviruses. Table S12. Nucleotide sequence identity of novel rodent and known coronaviruses (CoVs) in the RdRP-encoding region. Table S13. Nucleotide sequence identity of novel rodent and known hepeviruses (HEVs) in the ORF1 region. Table S14. Nucleotide sequence identity of novel rodent and known picornaviruses (PicoVs) in the RdRP-encoding region. Table S15. Nucleotide sequence identity of novel rodent and known astroviruses (AstroVs) in the RdRP-encoding region. Table S16. Nucleotide sequence identity of novel rodent unclassified RNA viruses and known viruses in the RdRP-encoding region.

**Additional file 2. Figure S10.** Phylogenetic tree based on the complete amino acid sequence of the RNA-dependent RNA polymerase of picornaviruses. The viruses found in this study are labeled in red font. Figure S11. Phylogenetic tree based on the complete amino acid sequence of the RNA-dependent RNA polymerase of astroviruses (AstroVs). The virus found in this study is labelled in red font.

## Data Availability

Datasets generated and analyzed during the current study are available in this published article (and its supplementary information files) and the NCBI sequence reads archive (SRA) under accession number PRJNA605875.
