# Supplementary material for: Decoding the RNA viromes in rodent lungs provides new insight into the origin and evolutionary patterns of rodent-borne pathogens in Mainland Southeast Asia
Source: Microbiome. 2021 Jan 21;9:18. doi: 10.1186/s40168-020-00965-z (PMC7818139; doi:10.1186/s40168-020-00965-z)
Supplement: Supplementary file 3 — Additional file 2. Figure S10. Phylogenetic tree based on the complete amino acid sequence of the RNA-dependent RNA polymerase of picornaviruses. The viruses found in this study are labeled in red font. Figure S11. Phylogenetic tree based on the complete amino acid sequence of the RNA-dependent RNA polymerase of astroviruses (AstroVs). The virus found in this study is labelled in red font. [file 40168_2020_965_MOESM3_ESM.docx]

**
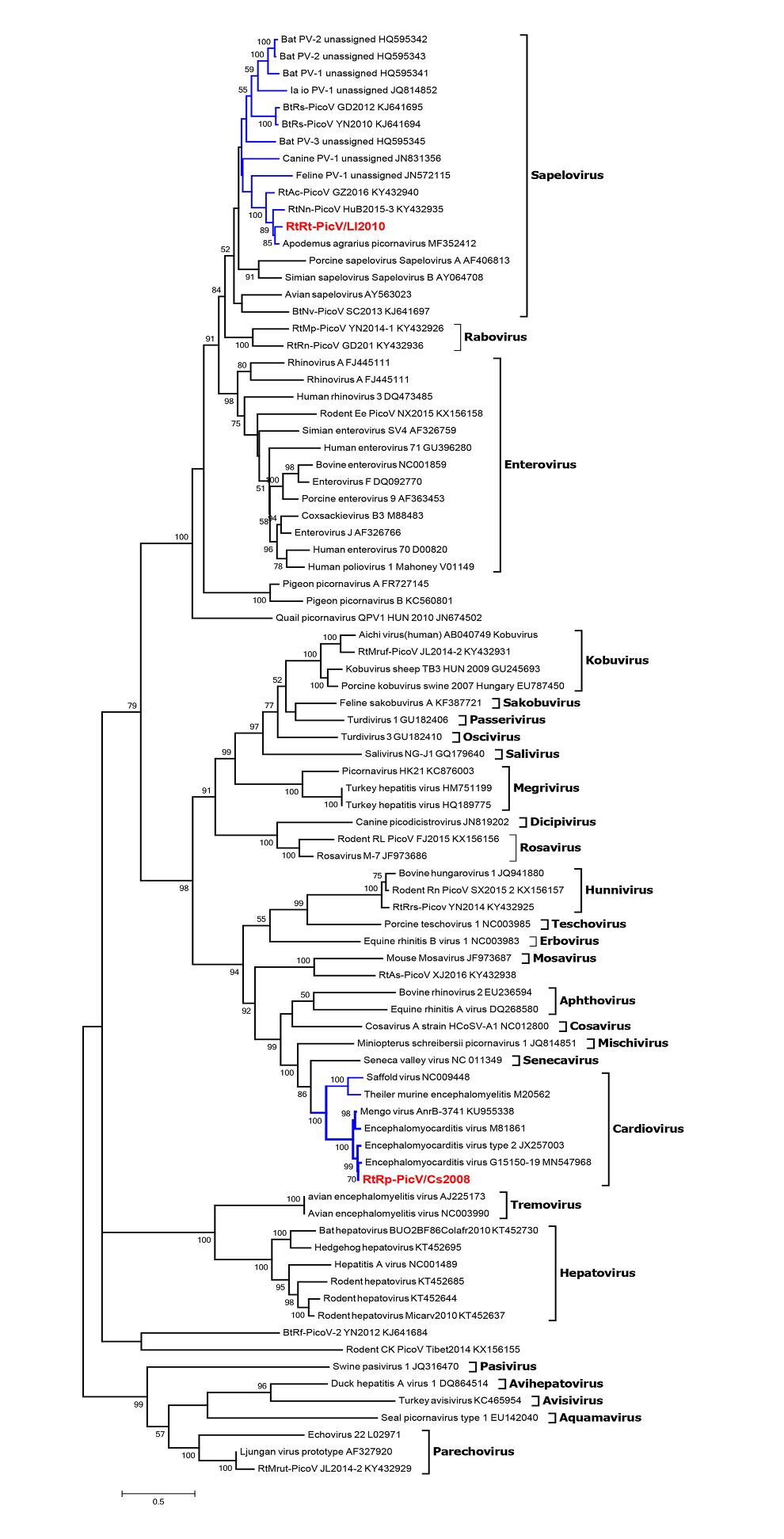
Figure S10.** **Phylogenetic tree based on the complete amino acid sequence of the RNA-dependent RNA polymerase of picornaviruses.** The viruses found in this study are labeled in red font.

**
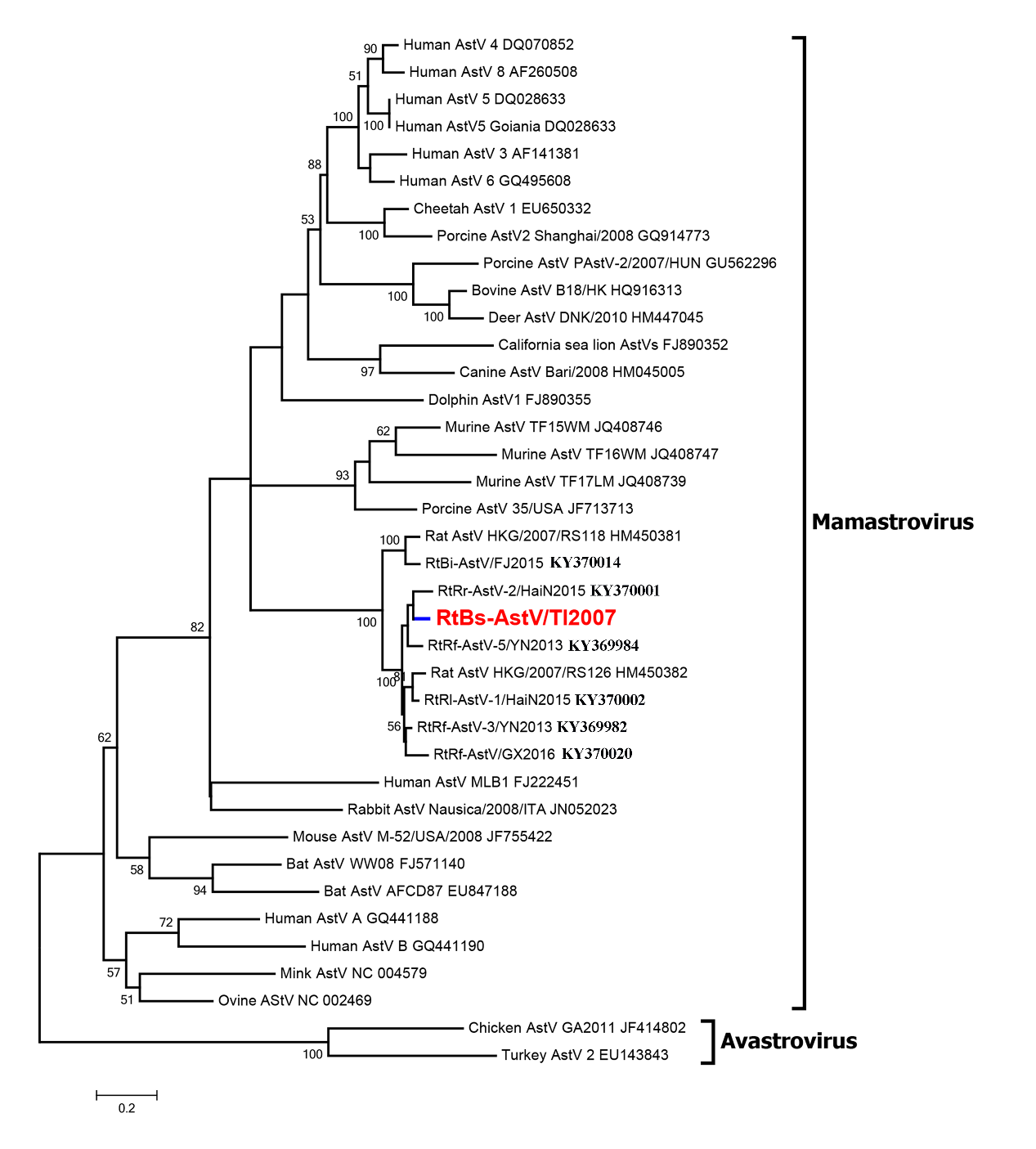
Figure S11.** **Phylogenetic tree based on the complete amino acid sequence of the RNA-dependent RNA polymerase of astroviruses (AstroVs).** The virus found in this study is labelled in red font.
